# Antioxidant Machinery Differs between Melanic and Light Nestlings of Two Polymorphic Raptors

**DOI:** 10.1371/journal.pone.0013369

**Published:** 2010-10-14

**Authors:** Ismael Galván, Laura Gangoso, Juan M. Grande, Juan J. Negro, Airam Rodríguez, Jordi Figuerola, Carlos Alonso-Alvarez

**Affiliations:** 1 Department of Evolutionary Ecology, Estación Biológica de Doñana (CSIC), Sevilla, Spain; 2 Department of Ecology and Evolution, Biophore, University of Lausanne, Lausanne, Switzerland; 3 Department of Biology, University of Saskatchewan, Saskatoon, Canada; 4 Department of Wetland Ecology, Estación Biológica de Doñana (CSIC), Sevilla, Spain; 5 Ecology Unit, Instituto de Investigación en Recursos Cinegéticos, IREC (CSIC, UCLM, JCCM), Ciudad Real, Spain; Arizona State University, United States of America

## Abstract

Colour polymorphism results from the expression of multiallelic genes generating phenotypes with very distinctive colourations. Most colour polymorphisms are due to differences in the type or amount of melanins present in each morph, which also differ in several behavioural, morphometric and physiological attributes. Melanin-based colour morphs could also differ in the levels of glutathione (GSH), a key intracellular antioxidant, because of the role of this molecule in melanogenesis. As GSH inhibits the synthesis of eumelanin (i.e. the darkest melanin form), individuals of darker morphs are expected to have lower GSH levels than those of lighter morphs. We tested this prediction in nestlings of two polymorphic raptors, the booted eagle *Hieraaetus pennatus* and the Eleonora's falcon *Falco eleonorae*, both of which occur in two morphs differing in the extent of eumelanic plumage. As expected, melanic booted eagle nestlings had lower blood GSH levels than light morph eagle nestlings. In the Eleonora's falcon, however, melanic nestlings only had lower GSH levels after controlling for the levels of other antioxidants. We also found that melanic female eagle nestlings had higher levels of antioxidants other than GSH and were in better body condition than light female eagle nestlings. These findings suggest an adaptive response of melanic nestlings to compensate for reduced GSH levels. Nevertheless, these associations were not found in falcons, indicating species-specific particularities in antioxidant machinery. Our results are consistent with previous work revealing the importance of GSH on the expression of melanic characters that show continuous variation, and suggest that this pathway also applies to discrete colour morphs. We suggest that the need to maintain low GSH levels for eumelanogenesis in dark morph individuals may represent a physiological constraint that helps regulate the evolution and maintenance of polymorphisms.

## Introduction

Genetic polymorphism can lead to the production of strikingly different phenotypes when the expression of a single gene depends upon the alleles present. This phenomenon is known as colour polymorphism when the main phenotypic difference is the colour of the plumage, pelage or skin, and has traditionally been defined as the existence of distinct, genetically determined colour morphs within a single interbreeding population, in such a proportion that the rarest form cannot be solely maintained by recurrent mutation [1]. The mechanisms responsible for the evolution and maintenance of colour polymorphisms in wild populations of animals have been the subject of intense research, especially in fish [2], reptiles [3] and birds [4].

Although the expression of colour polymorphisms depends weakly on environmental factors such as temperature or food [5; but see 6], their evolution and maintenance is determined by the action of non-genetic agents through natural selection [7], [8]. In birds for example, the maintenance of colour polymorphisms may be due to differential hunting success of colour morphs due to variation in light conditions [9]. However, selection does not necessarily have to act directly on the colour trait to generate differences in the adaptive value of each morph, as there are several physiological, morphometric and behavioural traits associated with each colour morph upon which natural selection could act as well [4], [10]. For example, if individuals of one colour morph have higher competitive ability or immunocompetence than those of the other morph, this would lead to viability selection against the latter [11], [12]. Consequently, pleiotropy in the genes responsible for colour expression would generate covariation between colour polymorphism and a number of individual attributes [10], [13].

An additional and, as far as we know, unexplored association between colour polymorphism and individual attributes can arise when the characteristics of these attributes depend on the biochemical basis of colour production, and not on pleiotropic effects of the genes related to colouration [e.g. 10], [14]. Most colour polymorphisms are directly due to changes in the proportion of areas covered by melanins in integumentary structures such as feathers, or in the type of melanin these structures contain [4], [13]. Therefore, the mechanisms that control the production of these pigments may be responsible for the causal relationship between colour polymorphism and some physiological attributes associated with melanogenesis [15]. Melanin pigments occur in two forms, with eumelanin being responsible for darker colours such as blacks and grey and pheomelanin responsible for lighter colours such as brown, red and orange [16].

Glutathione (GSH) is a tripeptide thiol found in virtually all animal cells that functions as the main physiological reservoir of cysteine [17] and as the most important intracellular antioxidant [18], [19]. GSH is also intrinsically involved in the process of melanin synthesis by melanocytes [20]–[22]. GSH influences melanogenesis by directly inhibiting the action of tyrosinase (the enzyme catalysing the first step of the process), by combating free radicals that stimulate the action of tyrosinase, and by increasing the ratio cysteine to dopaquinone [21]. Consequently, GSH levels determine the direction of melanogenesis: high tyrosinase activity and a low ratio of cysteine to dopaquinone lead to the production of eumelanin, while low tyrosinase activity and a high ratio of cysteine to dopaquinone lead to the production of pheomelanin, or even an absence of melanin synthesis [21], [23]. Therefore, low levels of GSH lead to eumelanogenesis while high levels lead to pheomelanogenesis, such that eumelanin production proceeds by default when antioxidant levels are low [22]. Since the vast majority of polymorphisms are expressed through variability in the content of eumelanin and pheomelanin in the integument of the individuals of the different morphs [e.g. 24]–[29], and eumelanin production occurs when GSH levels are low [20], [23], individuals with large eumelanin-based traits or a greater overall proportion of eumelanic integument may present a lower antioxidant capacity as a consequence of this expression mechanism [20], [21].

**Figure 1:**
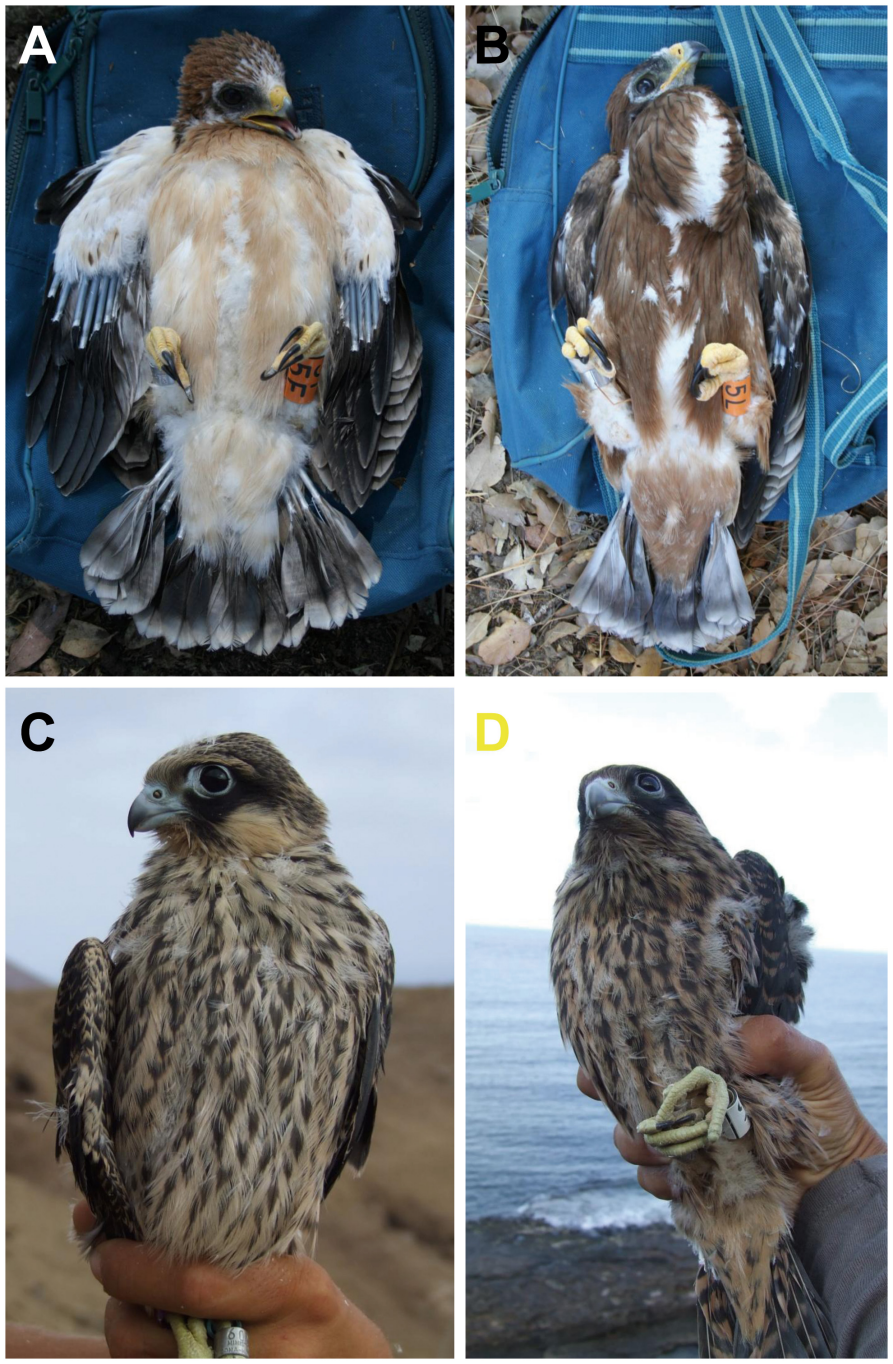
Booted eagle and Eleonora's falcon nestlings of different colour morphs. Examples of (A) light-morph and (B) melanic-morph booted eagle nestlings and of (C) light-morph and (D) melanic-morph Eleonara's falcon nestlings from the study populations.

We performed the first correlative test of the hypothesis that individuals of different melanin-based colour morphs differ in the levels of intracellular GSH during melanogensis, and consequently, in their antioxidant capacity. The study involved two species of birds of prey, the booted eagle Hieraaetus pennatus and the Eleonora's falcon Falco eleonorae. Birds of prey are ideal subjects for such studies as they are the group of birds in which colour polymorphisms are most common [30], [31]. Both the booted eagle and the Eleonora's falcon occur in two distinct morphs: the typical pale-coloured or ‘light’ morph and the less common dark or ‘melanic’ morph [sensu 13]. Adults and juveniles can be easily assigned to either morph based on plumage colouration [32]–[35], with melanic individuals having a greater amount of eumelanic feathers (Fig. 1). The plumage morphs of the Eleonora's falcon are inherited independently of gender in a Mendelian fashion, with melanic being the dominant allele [32]. The genetic basis of the booted eagle polymorphism is unknown. In both species, the melanic morph is less common than the light morph, as is the case in other polymorphic raptors [33,34, own unpublished data]. However, the species are phylogenetically distinct [36] and have very different life histories. Booted eagles, like most diurnal raptors, nest solitarily each spring and their breeding success depends upon the productivity of their territories [37]. In contrast, Eleonora's falcons breed colonially at the end of summer and time their reproduction to coincide with the southward migration of their avian prey, which can result in pronounced variation in food availability for their young [38, authors pers. obs.]. We chose to study both species to test for broad relationships between life-history features and measures of antioxidant machinery.

We tested the hypothesis by obtaining blood samples from wild nestlings of both species. We used nestlings because they have already started to show morph differences at that age, and because adults are difficult to catch. Furthermore, by using nestlings we avoided several confounding variables such as differences in experience between individuals, and reduced differences in age as compared to adults. We predicted that, in both species, nestlings of the melanic morph would have lower GSH levels than those of the light morph. Given the antioxidant properties of GSH [18], [19], we also predicted that nestlings from the melanic morph should maintain higher levels of alternative antioxidants than those of the lighter morph, which would protect them from oxidative damage incurred by their low GSH levels [20]. Therefore, in addition to comparing GSH levels between morphs, we also compared the concentration of alternative antioxidants, as estimated by the levels of uric acid and total antioxidant capacity of plasma, and also oxidative damage, as estimated by the level of lipid peroxidation in erythrocytes.

## Materials and Methods

### Ethics statement

All fieldwork was conducted with the required authorizations for capture, ringing and blood sampling of birds from the review boards of the Regional Government of the Canary Islands (Approval ID. 262/2009) and the Junta de Andalucía (Approval ID. 2628/MDCG/mect).

### Field methods

We sampled nestling booted eagles from territories monitored by the Natural Processes Monitoring Team of Doñana Biological Station in Doňana National Park in south west Spain (37°10′N, 6°23′W). 65 eagle nestlings were bled from 52 nests when they were 25–35 days old (34 nestlings from 28 nests in 2008 and 31 from 24 nests in 2009). Booted eagle nestlings at this age can easily be assigned to morphs because the ventral plumage is dark brown in the melanic morph but pale in the light morph (Fig. 1). We sampled Eleonora's falcon nestlings from a population of around 120 breeding pairs located in the Alegranza Islet on the north of Lanzarote, in the Canary Islands (27°37′–29°25′N, 13°20′–18°19′W) during September 2009. 132 falcon nestlings from 61 nests were bled when they were 25–30 days old (median  = 27.7 days old; their exact age in days was calculated by using the formula provided by [39]), and their colour morph was determined from the colour pattern of their undertail coverts, as even at this young age, birds of the melanic morph have dark brown undertail coverts while those of the light morph have pale coverts [40].

Blood samples (1 ml) were collected from the brachial vein and stored in a cooler in the field, then later centrifuged in the laboratory (within 6 hours of collection) and the plasma and red blood cells stored separately at −80°C until analysis.

### Determination of Total Glutathione Levels of Erythrocytes

Briefly, the blood pellet was weighed to the nearest 0.0001 g and thawed, and the red blood cells were drawn up with a pipette avoiding the pellet surface (i.e. the buffy coat containing white blood cells). Erythrocytes were immediately diluted (1∶10 w/v) and homogenized in a stock buffer [0.01 M phosphate buffered saline (PBS) and 0.02 M ethylene diamine tetra acetic acid (EDTA)], always working on ice to avoid oxidation. Three working solutions were made up in the same stock buffer as follows: (I) 0.3 mM nicotinamide adenine dinucleotide phosphate (NADPH), (II) 6 mM 5,5′-Dithiobis(2-nitrobenzoic acid) (DTNB), and (III) 50 Units of glutathione reductase/mL. An aliquot (0.5 mL) of red blood cell homogenate was vortexed with 0.5 mL of 10% trichloroacetic acid for 5 seconds thrice within a 15 minute period. Between each vortexing the samples were placed in a darkened refrigerator to prevent oxidation. Finally, the mixture as centrifuged at 1125 g for 15 minutes at 6°C and the supernatant removed and placed on ice. Subsequent steps were carried out in an automated spectrophotometer (A25-Autoanalyzer; Biosystems SA, Barcelona). Solutions 1 and 2 were mixed at a ratio of 7∶1 respectively and 160 uL of this mixture was added to 40 uL of the sample supernatant in a cuvette. After 15 seconds, 20 uL of solution 3 was added and then the absorbance at 405 nm was read after 30 and 60 seconds. The change in absorbance between the two readings was used to determine glutathione concentration in red blood cells according to a standard curve generated by serial dilution of glutathione from 1 mM to 0.031 mM. Repeatability of this technique was previously determined on a sub-sample of erythrocytes measured twice (*r* = 0.85, *n* = 20, *P* = 0.002). Concentration is presented as µmol glutathione/g of blood pellet.

### Determination of Lipid Peroxidation in Erythrocytes

The principle of the test is based on the fact that most tissues contain a mixture of thiobarbituric acid reactive substances (TBARS), including lipid hydroperoxides and aldehydes, whose concentrations increase due to oxidative stress. 1 mL of the homogenate used for the tGSH analysis was mixed with 2 mL of a solution of 15% trichloroacetic acid, 0.77% hydrochloric acid and 0.375% thiobarbituric acid and with 20 uL of 2% 2,6-di-tert-butyl-4-methylphenol (BHT) in ethanol in a closed glass tube. Tubes were then heated for 30 min at 90°C and then cooled in ice-cold water for 10 min. The mixture was centrifuged at 2025 g for 15 min and the absorbance of the supernatant was read at 535 nm. The concentration of peroxidised lipids was determined in reference to a standard curve with 0, 1.25, 2.5 and 5 nmol/mL of malondialdehyde (MDA) in H_2_O (i.e. end product of lipid peroxidation) and presented as nM MDA/g of red blood cell pellet. Repeatability was previously estimated on a subset of samples measured three times (*r* = 0.80, *n* = 20, *P* = 0.037).

### Determination of Plasma Uric Acid Concentration and Antioxidant Capacity

Uric acid concentration of plasma was determined in a Cobas Integra 400 Automated Analyser using the commercial enzymatic colorimetric method (Roche Diagnostics S.L., San Cugat del Vallés, Barcelona, Spain). Total Antioxidant Capacity (TAC) of plasma was determined through a colorimetric assay. This method is based upon the colour change, adapted from [41], caused by the addition of hydrogen peroxide to colourless 2,2V-azinobis(3-ethylbenzo-thiazoline-6-sulfonate) (ABTS), which oxidizes it into a characteristic blue-green solution. 5 µl plasma was mixed with 200 µl 0.4 M acetate buffer (pH 5.8) and 30 µl of a solution of 30 mM acetate buffer, 2 mM H_2_O_2_ and 10 mM ABTS. Absorbance was measured every 30 seconds for 10 minutes at 595 nm on a 1420 Multilabel Counter Victor 3 (Perkin Elmer), and antioxidant capacity was measured as the rate of change of absorbance. TAC was estimated as the average of two replicate measurements per individual. Distilled water was used as a blank and TAC was calibrated with a standard of Trolox (an hydrosoluble equivalent of Vitamin E) and expressed in mM Trolox equivalent. The repeatabiity of these techniques was determined on a subset of samples measured two times (uric acid concentration: *r* = 0.99, *n* = 10, *P*<0.0001; TAC: *r* = 0.89, *n* = 10, *P*<0.0001).

### Molecular Sexing

Nestling eagles and falcons were sexed using DNA extracted from blood, which was amplified by PCR using the primers 2550F and 2718R following Fridolfsson and Ellegren [42].

### Statistical Analyses

Generalized Linear Mixed Models (GLMMs) were used to determine the predictors of total glutathione and lipid peroxidation levels in erythrocytes and TAC and uric acid levels in plasma of Eleonora's falcon and booted eagle nestlings. All these variables were dependent variables in single GLMMs, and morph (light vs. melanic) and sex were added as fixed factors. Brood size was added as a covariate, because this may be positively correlated with oxidative stress levels [43], [44]. We also included body mass as a covariate, as this could affect oxidative stress, with nestlings that develop more rapidly suffering greater stress [44]. In the case of Eleonora's falcons, we included body mass and estimated age as covariates. In every model, levels of other antioxidants were added as covariates to understand the pattern of covariation between them [e.g. 45]. We accounted for the common origin of nestlings and year (in the case of booted eagles) by adding nest identity and year as random factors, and the model was fitted using restricted maximum likelihood (REML).

Unfortunately, some data on body mass and tarsus length of booted eagles in 2008 was lost during a hardware accident. The remaining data were thus analysed separately to avoid a decrease in statistical power when analysing the effect of interest (i.e. colour morph) with the complete dataset. Hence the results from the booted eagle models with and without the morphometric data are presented separately (2008: n = 10 for body mass, n = 0 for tarsus length; 2009: n = 27 for both body mass and tarsus length).

In all models, we included an interaction between morph and sex to test whether there are sex-specific associations between morph and antioxidant levels and oxidative stress. In addition to the independent variables described above, the models also included two parameters to control for potential variability during the laboratory analyses of tGSH and TBARS. First, we included the weight of the blood pellet before it was homogenized. Second, the Eleonora's falcon samples were analysed in three separate assays because of the larger sample size. The assay in which each sample was analysed was therefore added as a random factor.

Finally, we analysed variability in nestling body condition using models where body mass was the dependent variable and tarsus length was included as a covariate to control for body size [46]. The other predictor variables were those used in the models examining levels of antioxidants and oxidative damage.

We removed non-significant terms from each saturated model using a backward elimination procedure with a *P*-value of 0.1 being sufficient to eliminate the term. Random factors were always maintained in the models, and in all cases, the distribution of the residuals confirmed that the assumption of normality was fulfilled. Satterthwaite correction was used to approximate the denominator degrees of freedom.

## Results

### Booted Eagles

#### Total glutathione levels

**Figure 2:**
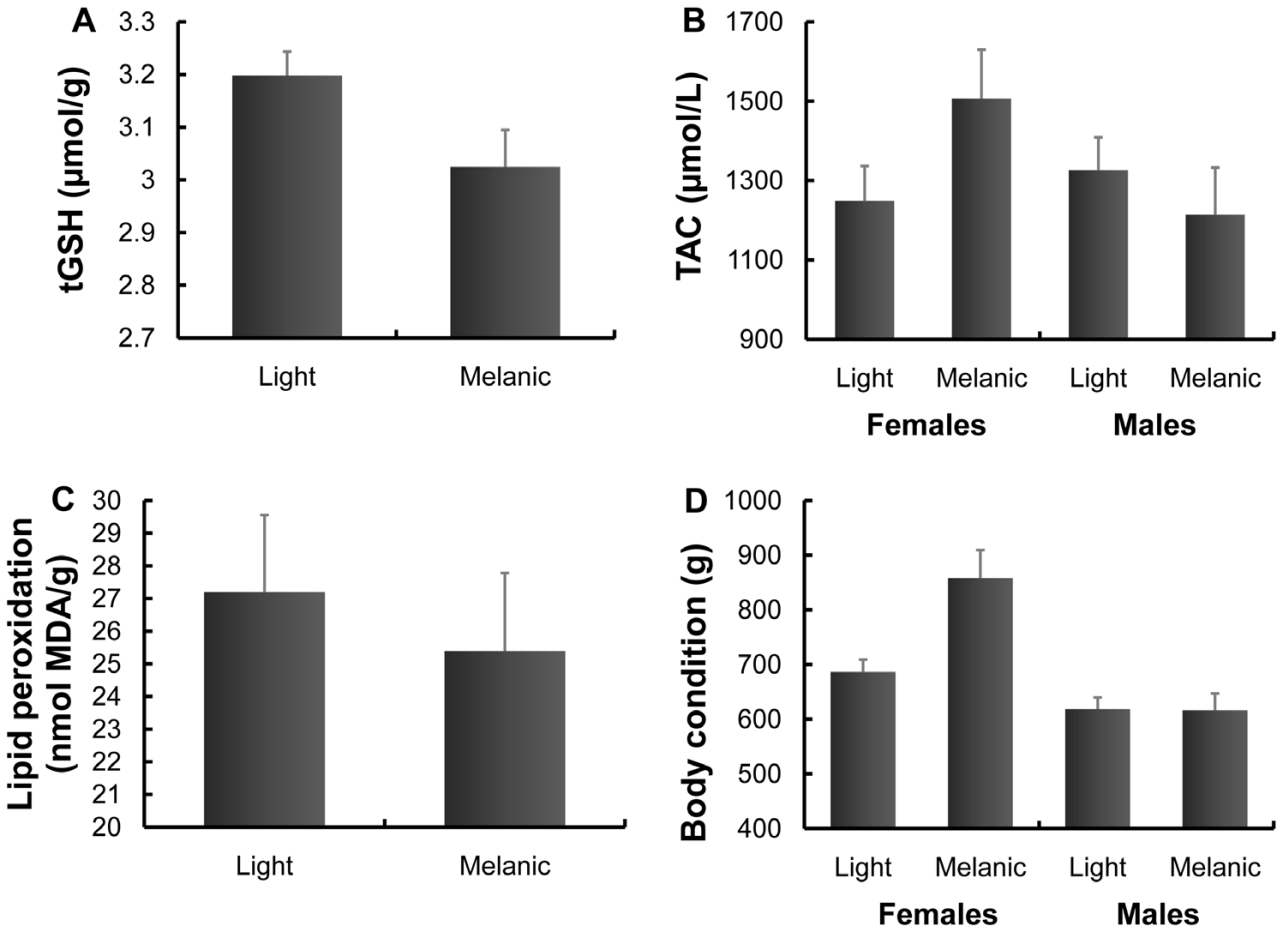
Physiological parameters of light and melanic morphs of nestling booted eagles of each sex. (A): Total glutathione (tGSH) level in pelleted erythrocytes. (B): Plasma total antioxidant capacity (TAC). (C): Lipid peroxidation level of pelleted erythrocytes. (D): Body condition (mass corrected for body size). Least squares means + standard error are shown.

There was a significant difference in glutathione levels between nestlings of the two colour morphs (Table 1), with those of the melanic morph having lower levels than those of the light morph (Fig. 2A). Considering the subsample of individuals where morphometric data were available, nestling mass was not significantly related to tGSH when added to the previous model (*F*_1,29.3_ = 1.93, *P* = 0.175).

**Table 1:** Results of the models explaining variability in total glutathione (tGSH) levels in erythrocytes and plasma antioxidant levels (TAC and uric acid) in booted eagle and Eleonora's falcon nestlings.

Booted eagle												
	tGSH				TAC				Uric acid			
*Effect*	*b*	*F/Z*	*df*	*P*	*b*	*F/Z*	*df*	*P*	*b*	*F/Z*	*df*	*P*
Morph	-	**4.33**	**1,53**	**0.042**	-	0.01	1,21.1	0.908	-	-	-	-
Sex	-	-	-	-	-	**4.97**	**1,16.7**	**0.040**	-	-	-	-
Morph x Sex	-	-	-	-	-	**5.09**	**1,13.8**	**0.040**	-	-	-	-
Body mass	-	-	-	-	-	-	-	-	-	-	-	-
Tarsus length	-	-	-	-	-	-	-	-	-	-	-	-
Nest identity	-	-	-	-	-	**3.06**	**-**	**0.001**	**-**	**3.96**	**-**	**<0.0001**
Year	-	-	-	-	-	0.20	-	0.420	-	-	-	-
tGSH	-	-	-	-	**440.93**	**11.05**	**1,15.6**	**0.004**	**3.64**	**5.68**	**1,16.3**	**0.030**

Eleonora's falcon												
Morph	-	0.00	1,120	0.952	**-**	**-**	**-**	**-**	**-**	**-**	**-**	**-**
Sex	-	0.48	1,107	0.492	**-**	**-**	**-**	**-**	**-**	**-**	**-**	**-**
Morph x Sex	**-**	**4.74**	**1,102**	**0.032**	**-**	**-**	**-**	**-**	**-**	**-**	**-**	**-**
Body mass	**3.24**×**10^−3^**	**21.74**	**1,118**	**<0.0001**	**−1.59**	**5.76**	**1,85.1**	**0.019**	**-**	**-**	**-**	**-**
Age	**-**	**-**	**-**	**-**	**-**	**-**	**-**	**-**	**-**	**-**	**-**	**-**
Tarsus length	-	-	-	-	**-**	**-**	**-**	**-**	**-**	**-**	**-**	**-**
Nest identity	**-**	**2.87**	**-**	**0.002**	**-**	**1.74**	**-**	**0.041**	**-**	**3.45**	**-**	**<0.001**
Homogenate mass	**−4.70**	**27.57**	**1,111**	**<0.0001**	**-**	**-**	**-**	**-**	**-**	**-**	**-**	**-**
Laboratory assay number	-	0.90	-	0.183	**-**	**-**	**-**	**-**	**-**	**-**	**-**	**-**
tGSH	-	-	-	-	**291.21**	**14.25**	**1,89.9**	**<0.001**	**0.90**	**3.39**	**1,106**	**0.068**

In the same model, but including alternative antioxidants as covariates, only TAC was negatively correlated with tGSH. However, the trend was non-significant (*b* = −1.9×10^−4^, *F*_1,50_ = 3.54, *P* = 0.066) and did not change the significant difference in tGSH between morphs (*F*_1,50_ = 4.49, *P* = 0.039).

#### Plasma antioxidants

In the model testing for variability in TAC, the only factor that was significant was uric acid concentration (*b* = 57.86, *F*_1,51_ = 113.92, P<0.0001), although it did not differ between morphs (see below). However, when uric acid concentration was not considered, tGSH remained in the model, as well as morph, sex (least square mean (LSM) ± s.e.: males: 1224.10±76.74 µmol/g, females: 1399.89±80.95 µmol/g), nest identity, year and the interaction between morph and sex (Table 1). This interaction was due to melanic females having significantly higher TAC levels than light females, whereas there was no difference in TAC levels between males of the two morphs (Fig. 2B). There was no effect of nestling body mass (*F*_1,10.8_ = 0.12, *P* = 0.732). Moreover, if tGSH and uric acid levels were not included in the TAC model as covariates, the interaction between morph and sex remained marginally significant (interaction: *F*_1,30_ = 3.75, *P* = 0.062; morph: *F*_1,43_ = 0.50, *P* = 0.484; sex: *F*_1,32.1_ = 1.27, *P* = 0.268; Fig. 2B). Nest identity also had a significant effect (Z = 2.25, P = 0.012), although year did not (Z = 0.05, P = 0.482).

The only significant terms in the model for uric acid levels were TAC (*b* = 0.01, *F*_1,47.8_ = 90.19, *P*<0.0001) and nest identity (Z = 1.78, P = 0.037). When TAC was not included, only tGSH and nest identity remained in the model (Table 1). Body mass did not affect uric acid levels (*F*_1,18.4_ = 0.12, *P* = 0.738).

#### Oxidative damage

The difference in the levels of oxidative damage (TBARS) between melanic and light nestlings (LSM ± s.e.: melanic morph: 25.62±2.17 nmol MDA/g, light morph: 26.89±2.11 nmol MDA/g) was not significant (*F*_1,48_ = 2.80, *P* = 0.100). In this model, there was also an effect of sex, though not of nest identity and year (Table 2). However, the difference in TBARS between the colour morphs became significant (*F*_1,8.01_ = 7.23, *P* = 0.027; Fig. 2C) after controlling for body mass by including it as a covariate in the previous model (*b* = 4.16×10^−3^, *F*_1,11.8_ = 1.78, *P* = 0.208; sex: *F*_1,8.26_ = 19.99, *P* = 0.002; nest identity: *Z* = 2.33, *P* = 0.010; year: *Z* = 0.68, *P* = 0.249).

**Table 2:** Results of the models explaining variability in oxidative damage (TBARS) levels of erythrocytes and body condition in booted eagle and Eleonora's falcon nestlings.

Booted eagle								
	TBARS				Body condition			
*Effect*	*b*	*F/Z*	*df*	*P*	*b*	*F/Z*	*df*	*P*
Morph	-	-	-	-	**-**	**8.89**	**1,12.6**	**0.011**
Sex	-	3.47	1,42.6	0.069	**-**	**14.75**	**1,23**	**<0.001**
Morph x Sex	-	-	-	-	**-**	**7.46**	**1,18.5**	**0.013**
Body mass	-	-	-	-	-	-	-	-
Tarsus length	-	-	-	-	**-**	**15.85**	**1,21.2**	**<0.001**
Nest identity	-	0.23	-	0.409	-	1.44	-	0.075
Year	-	0.69	-	0.246	-	-	-	-
tGSH	-	-	-	-	-	-	-	-

Eleonora's falcon								
Morph	-	-	-	-	-	-	-	-
Sex	-	-	-	-	**-**	**21.83**	**1,71.1**	**<0.0001**
Morph x Sex	-	-	-	-	-	-	-	-
Body mass	**−0.02**	**5.87**	**1,70.8**	**0.018**	-	-	-	-
Age	0.26	3.90	1,71	0.052	**11.84**	**112.37**	**1,93.1**	**<0.0001**
Tarsus length	-	-	-	-	**7.09**	**5.49**	**1,87.4**	**0.021**
Nest identity	**-**	**2.03**	**-**	**0.021**	**-**	**4.35**	**-**	**<0.0001**
Homogenate mass	−15.96	3.84	1,80.3	0.053	-	-	-	-
Laboratory assay number	-	0.99	-	0.160	-	-	-	-
tGSH	**2.21**	**8.04**	**1,83.1**	**0.006**	10.21	3.05	1,87.8	0.084
TAC	−1.5×10^−3^	3.10	1,82.9	0.082	-	-	-	-

#### Body condition

Body condition was significantly related to morph, sex, tarsus length and nest identity (Table 2). However, there was also a significant morph by sex interaction (Table 2), such that melanic females were in better condition than the other three groups (Fig. 2D).

### Eleonora's falcons

#### Total glutathione

**Figure 3:**
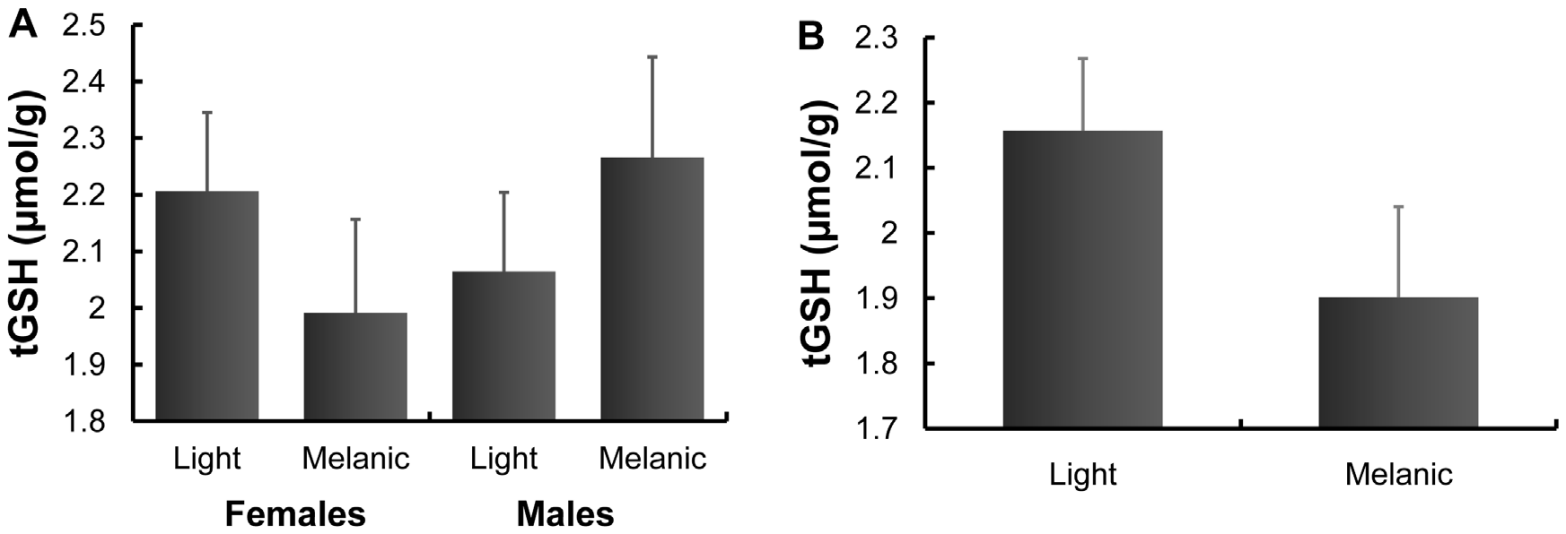
Total glutathione (tGSH) levels in male and female Eleonora's falcon nestlings of light and melanic morphs. Least squares means + standard error from models that (A) did not control for the variability in other antioxidants (total antioxidant capacity and uric acid) and that (B) included these variables as covariates are shown.

tGSH levels also differed between colour morphs of the Eleonora's falcon, but in this species the difference depended upon the sex of the nestling. The model thus included sex, morph and their interaction (Table 1). Melanic male nestlings had higher tGSH levels than light males, though melanic female nestlings had lower tGSH levels than light females (Fig. 3A). There was no difference between tGSH levels of light morph females and melanic males (post-hoc test: *P* = 0.690), but both had higher levels than light morph males and melanic females (*P* = 0.030), between which there was no difference in tGSH level (*P* = 0.590). Other terms in the model were nestling mass, homogenate mass, nest identity and laboratory assay number (Table 1).

When variability in all antioxidants was examined by adding TAC and uric acid levels as covariates, tGSH levels differed between morphs independently of their sex (*F*_1,72.1_ = 5.16, *P* = 0.026), with melanic falcons having lower tGSH levels than light morph falcons (Fig. 3B). The sex and sex x morph interaction were not significant (both P>0.3). In this species, however, TAC (*F*_1,71.1_ = 5.61, *P* = 0.020) and uric acid levels (*F*_1,68.6_ = 11.49, *P* = 0.001) covaried positively with tGSH (*b* = 2.56×10^−4^ and 0.05, respectively). Other variables remaining in the model were body mass (*b* = 3.95×10^−3^, *F*_1,72.8_ = 31.23, P<0.0001), homogenate mass (*b* = −4.35, *F*_1,71_ = 22.04, *P*<0.0001), nest identity (*Z* = 2.24, *P* = 0.013) and laboratory assay (*Z* = 0.84, *P* = 0.199).

#### Plasma antioxidants

TAC levels did not differ between morphs, and the model only included the effects of nestling age (*b* = −24.10, *F*_1,78.6_ = 3.22, *P* = 0.077) and uric acid (*b* = 32.25, *F*_1,63.2_ = 4.56, *P* = 0.037; nest identity: *Z* = 1.22, *P* = 0.110). When uric acid was not considered, the final model included nestling body mass, tGSH and nest identity (Table 1).

The model for uric acid levels only included the effects of tGSH (Table 1). The same result was obtained when TAC was not considered in the model.

#### Oxidative damage

TBARS levels did not differ between morphs. The model included nestling age, body mass, TAC levels, tGSH, homogenate mass, nest identity and laboratory assay number (Table 2).

#### Body condition

The body condition of Eleonora's falcon nestlings did not differ between morphs (*F*_1,42.1_ = 0.01, *P* = 0.935), and only nestling age, sex (LSM ± s.e.: males: 371.85±7.32 g, females: 401.24±7.05 g), tarsus length, tGSH and nest identity remained in the model (Table 2).

## Discussion

The present study adds to the relatively few reports of physiological differences between colour morphs [14; see however review in 4] and, as far as we know, is the first study reporting that polymorphism covaries with levels of antioxidants and oxidative damage.

Based upon previous findings in other bird species without colour polymorphism [i.e. 20], we predicted that individuals of the melanic morph of both species should have lower GSH levels than individuals of the light morph, and that individuals of the melanic morph should compensate for the low GSH levels required for eumelanogenesis by increasing the level of alternative plasmatic antioxidants. Although our results were consistent with both hypotheses, there were several important differences between species and sex, and some key interactions between the two. First, whereas there were significant differences in GSH levels between morphs of both sex in the booted eagle, the direction of the difference depended on sex in the Eleonora's falcon: melanic males had higher levels than light males, while melanic females had lower levels than light females. Second, the correlation between GSH and alternative antioxidants was negative in eagles but positive in falcons. Third, female booted eagle nestlings of the melanic morph had higher total antioxidant capacity than females of the light morph. Thus, female eagle nestlings of the melanic morph had lower GSH levels but also higher levels of total antioxidant capacity than those of the light morph. Moreover, melanic eagles showed lower mean values of oxidative damage than light eagles, although the difference was non-significant, which suggests the existence of a compensatory mechanism [e.g. 47], at least in this species.

The results showed both sex-specific and species-specific differences between morphs in their levels of circulating antioxidants and body condition. Morph was not related to plasma antioxidants or body condition in Eleonora's falcons, despite a larger sample size, which suggests there is no compensatory antioxidant mechanism in this raptor species. However, melanic falcons did not have higher levels of oxidative damage than light falcons, which suggests that they may have more efficient antioxidant machinery. Moreover, the lower levels of GSH in melanic falcons of both sexes were only apparent after controlling for variation in the levels of other antioxidants, suggesting that this species possesses a modified or attenuated role for GSH in melanin synthesis.

Species-specific associations between colour polymorphism and antioxidant levels may result from pleiotropic effects of different genes regulating colour production in each species. Probably the most important gene in this context is the pro-opiomelanocortin (POMC) gene, which codes for the production of several molecules with pleiotropic effects [see 10]. These molecules are named melanocortins, which are peptidic compounds that bind to melanocortin receptors. Although several melanin-based colour polymorphisms in wild birds depend on the expression of the melanocortin-1 receptor gene [MC1R; 8,26,48–51], a number of other genes coding for receptors of other different melanocortins are also involved in melanin production [51]. Thus, differences between species in the number and/or identity of genes that influence the expression of melanin-based polymorphism may result in different patterns of covariation between colour and levels of plasma antioxidants and oxidative damage.

Furthermore, the life history differences between the two species likely expose them to different environmental factors, which may in turn influence their levels of oxidative stress. For example, Eleonora's falcons breed colonially in late summer while booted eagles breed as territorial pairs in early spring. Coloniality is known to be strongly associated with the prevalence of haematophagous ectoparasites and haematozoan infection [52], and the latter of these has been shown to generate oxidative stress [53]. Differences in the amount and regularity of food delivered to the nestlings of each species may also explain the variation between morphs in levels of plasma antioxidants and oxidative stress. In booted eagles, the total amount of food nestlings receive will depend upon the productivity of their territory, but in general they deliver prey to the brood on a regular basis [37]. In contrast, falcons prey upon migratory birds. Since the number of migrating birds fluctuates according to the prevailing wind conditions [38, authors pers. obs.], the nestlings experience a very erratic food supply, which likely causes sharp changes in physiological stress and in uric acid levels [e.g. 54], [55], that may unbalance their redox homeostasis. This may have resulted in different associations of colour morph with the levels of plasma antioxidants and oxidative damage in Eleonora's falcons and booted eagles. Additional factors such as geographic location, the different breeding periods (autumn in Eleonora's falcons vs. spring-summer in booted eagles) or diet composition (exclusively birds in the falcons and including other vertebrates in the eagles) may also be important.

Our results show that morphs differing in the eumelanin content of their plumage also differ in their antioxidant machinery. We suggest that these differences are a direct consequence of the central role of GSH in eumelanin production [20]–[22], rather than the result of pleiotropic genes affecting both melanization and antioxidant levels [10]. This would mean that a melanin-based colour polymorphism may constrain the antioxidant levels of the individuals that produce the melanic morph, which does not exclude the possibility that pleiotropic effects on colour are also present.

Given the importance of oxidative stress in determining life-history traits [reviewed in 56], there will likely be fitness consequences for the individuals of each morph. It is worth mentioning that the melanic morph of the Eleonora's falcon seems to be much less often observed in nature [32]–[34], and the same tendency is observed in other polymorphic raptors [35]. Indeed, our results show that melanic booted eagle of any sex, and female melanic Eleonora's falcons, have lower GSH levels than conspecifics of the light morh. It is possible that morph frequencies are maintained by frequency-dependent selection, so that light individuals may present fitness disadvantages of the same magnitude than that represented by low GSH levels in melanic individuals, resulting in a ratio costs:benefits that is similar in both morphs [see [57] for an example of positive selection on darker individuals]. However, there is support for fitness disadvantages or at least exceptionally low observation and capture frequencies in the more eumelanized morph unrelated to direct natural selection pressures on colour (e.g. predation or thermoregulation) in other bird species [58; see however 12] and also in mammals [59,60] and insects [15]. Some reports indicate that birds of the more eumelanized morph are more vulnerable to bacterial infections [61] and ectoparasites [27; but see 62]. Therefore, perhaps individuals belonging to the melanic morph pay a fitness cost, despite the lower oxidative damage that we observed in melanic nestling booted eagles as compared to light nestlings. The lower oxidative damage found in melanic booted eagles as compared to light eagles may occur because of the adaptive response to compensate for the low GSH levels required for eumelanogenesis may be physiologically costly, or because this adaptive response does not always occur. Another possibility is that this adaptive response can be only performed by individuals of high genotypic quality, whereas low quality individuals are not able to make the antioxidant compensation and suffer physiological consequences after trying to maintain low GSH levels. Future studies should explore these possibilities.
